# Thiamine-modified metabolic reprogramming of human pluripotent stem cell-derived cardiomyocyte under space microgravity

**DOI:** 10.1038/s41392-024-01791-7

**Published:** 2024-04-08

**Authors:** Xinglong Han, Lina Qu, Miao Yu, Lingqun Ye, Liujia Shi, Guangfu Ye, Jingsi Yang, Yaning Wang, Hao Fan, Yong Wang, Yingjun Tan, Chunyan Wang, Qi Li, Wei Lei, Jianghai Chen, Zhaoxia Liu, Zhenya Shen, Yinghui Li, Shijun Hu

**Affiliations:** 1 Department of Cardiovascular Surgery of the First Affiliated Hospital & Institute for Cardiovascular Science, State Key Laboratory of Radiation Medicine and Protection, Suzhou Medical College, Soochow University, Suzhou, China; 2 State Key Laboratory of Space Medicine, China Astronaut Research and Training Center, Beijing, China; 3 Department of Hand Surgery, Union Hospital, Tongji Medical College, Huazhong University of Science and Technology, Wuhan, China

**Keywords:** Cardiology, Pluripotent stem cells

## Abstract

During spaceflight, the cardiovascular system undergoes remarkable adaptation to microgravity and faces the risk of cardiac remodeling. Therefore, the effects and mechanisms of microgravity on cardiac morphology, physiology, metabolism, and cellular biology need to be further investigated. Since China started constructing the China Space Station (CSS) in 2021, we have taken advantage of the Shenzhou-13 capsule to send human pluripotent stem cell-derived cardiomyocytes (hPSC-CMs) to the Tianhe core module of the CSS. In this study, hPSC-CMs subjected to space microgravity showed decreased beating rate and abnormal intracellular calcium cycling. Metabolomic and transcriptomic analyses revealed a battery of metabolic remodeling of hPSC-CMs in spaceflight, especially thiamine metabolism. The microgravity condition blocked the thiamine intake in hPSC-CMs. The decline of thiamine utilization under microgravity or by its antagonistic analog amprolium affected the process of the tricarboxylic acid cycle. It decreased ATP production, which led to cytoskeletal remodeling and calcium homeostasis imbalance in hPSC-CMs. More importantly, in vitro and in vivo studies suggest that thiamine supplementation could reverse the adaptive changes induced by simulated microgravity. This study represents the first astrobiological study on the China Space Station and lays a solid foundation for further aerospace biomedical research. These data indicate that intervention of thiamine-modified metabolic reprogramming in human cardiomyocytes during spaceflight might be a feasible countermeasure against microgravity.

## Introduction

With the constant development of space technology, human space exploration in low-earth orbit, the moon, and beyond will become more commonplace. However, exposure to space microgravity, which differs from the normal state of gravity, could trigger adaptation and lasting effects on the human body, including multi-organ degeneration, dysfunction, abnormal structure, metabolic disorder, premature senescence, and so on.^1–4^ For the cardiovascular system, astronauts on space shuttle missions experience reduced heart rate, lowered arterial pressure, arrhythmias, cardiac atrophy, anemia, and other aging-like deconditioning, such as loss of physical fitness, arterial stiffening, and development of insulin resistance.^5,6^ However, there is still a lack of effective treatments and prevention methods to alleviate the symptoms.

Because of the critical role of the heart in maintaining proper bodily systemic functions, a series of studies have been carried out in rodent and cell models to investigate the effects of microgravity on cardiac physiology, metabolism, and cellular biology.^7–10^ In Drosophila, microgravity reduces heart size, contractility, and proteostasis imbalance.^11^ The potential mechanisms underpinning adverse cardiac dysfunction or adaptation in response to microgravity include decreased metabolism, alerted calcium handling, increased oxidative stress, inflammation, and apoptosis.^6^ Despite the above studies, the mechanisms underlying cardiac abnormalities in a microgravity environment are incompletely understood, and the countermeasures need to be further explored.

The heart is a highly energy-consuming organ. For cardiomyocyte contraction and relaxation to occur, many ATPs are consumed during cytoskeleton assembly and rearrangement, ATPase-driven calcium pumping, and myofilament sliding.^12^ In cardiomyocytes, mitochondrial oxidative metabolism is the most significant contributor to ATP production.^13^ As for cardiac metabolism, substrate conversion and energetic deficiency may lead to contractile dysfunction and the progression of heart disease.^14,15^ Previous studies showed significant changes in mitochondrial metabolic pathways in both rat hearts and human cardiomyocytes after microgravity exposure, yet the detailed mechanisms are still not understood.^16,17^ Thiamine, also known as vitamin B1, is involved in the metabolism of both carbohydrates and amino acids, acting as the coenzyme of pyruvate dehydrogenase complex, α-ketoglutarate dehydrogenase complex, and transketolase.^18^ Thiamine deficiency could reduce tricarboxylic acid (TCA) cycle efficiency and impair ATP production.^19,20^ Thiamine supplementation has been proven as an effective way to reduce risk and improve outcomes in the cardiovascular system.^21^ However, there is still a lack of related studies about thiamine metabolism in the space environment.

Due to the inaccessibility of human cardiomyocytes and limited space resources, most mechanism investigations were based on rodent models in the simulated microgravity conditions on Earth. Human PSCs, including human embryonic stem cells (hESCs) and human induced pluripotent stem cells (hiPSCs), have shown a strong potential for cardiomyocyte differentiation in vitro. Recently, Dr. Wu’s group indicated that hiPSC-derived cardiomyocytes (hiPSC-CMs) could model the effects of microgravity on the human heart and identified the alterations in calcium handling and gene expression at the cellular level during spaceflight on the International Space Station.^16^ Thus, hPSC-CMs represent an ideal source of human cardiomyocytes to study the effects of microgravity on cell-level cardiac function.

The China Space Station (CSS) is a long-term space station, orbiting between 340 and 450 kilometers above the Earth’s surface. The station serves as a space environment research laboratory in which scientific researches are conducted in astrobiology, astronomy, physics, and other fields. Since China started constructing the CSS in 2021, we took advantage of the Shenzhou-13 (SZ-13) capsule launched on October 16, 2021, and carried out the first biological experiment in the Tianhe core module on the CSS. We investigated the effects of space microgravity on hPSC-CMs via dynamically recording the abnormal cell contraction and calcium transient fluorescence signal in cardiomyocytes aboard the CSS. High-throughput metabolomic and transcriptomic analyses indicated the impaired thiamine utilization in cardiomyocytes under space microgravity conditions. Supplementing thiamine in cardiomyocytes and mice could rescue the defects caused by microgravity.

## Results

### The preparation of hPSC-CMs for the SZ-13 space mission

**Fig. 1:**
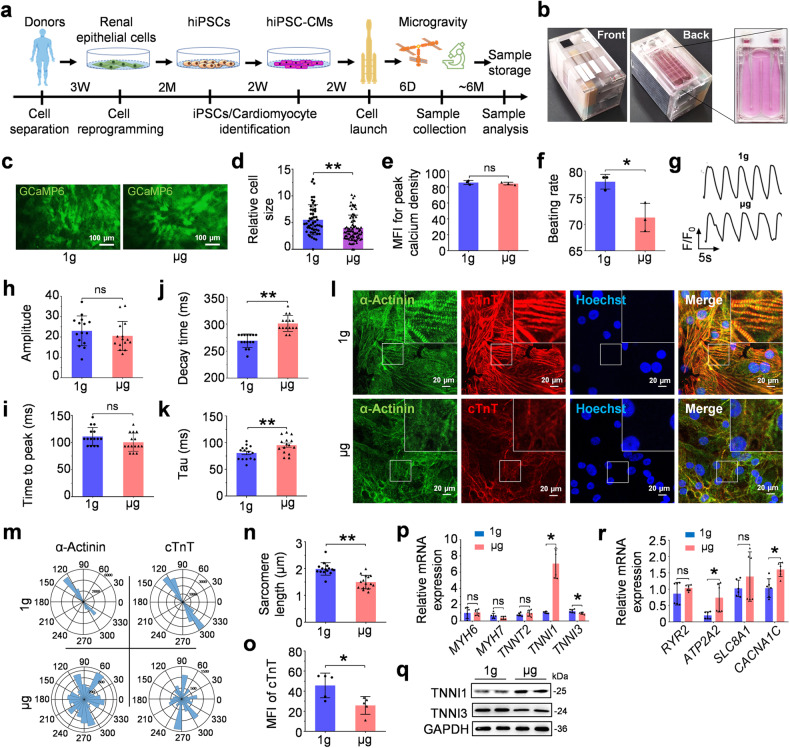
Space microgravity leads to cardiomyocyte adaptative changes. **a** A schematic of all experimental processes, including somatic reprogramming, cardiomyocyte preparation, and cell treatment during the SZ-13 mission. **b** The representative pictures of culture flasks and apparatus for cell maintenance on the CSS. **c** The representative images of GCaMP-CMs under space microgravity (μg) and simulated 1 g (1g) conditions on the CSS. **d** The quantitative analyses on cell size of GCaMP-CMs in 1 g and μg groups. **e** The quantitative comparison on MFI (mean fluorescence intensity) for peak calcium density of GCaMP-CMs under 1 g and μg conditions. **f** The beating rate analyses of GCaMP-CMs under 1 g and μg conditions. **g** Representative calcium transients of GCaMP-CMs. The quantitative analyses of amplitude (**h**), time to peak (**i**), decay time (**j**) and tau (**k**) of GCaMP-CMs under 1 g and μg conditions. **l** Immunofluorescence staining of cytoskeleton proteins in hPSC-CMs under 1 g and μg conditions (α-Actinin, green; cTnT, red; Hoechst 33342, blue). **m** Representative polar histogram of myofibrillar alignment in hPSC-CMs under 1 g and μg conditions. The quantitative analyses on sarcomere length (**n**) and cTnT content (**o**) under 1 g and μg conditions. **p** The relative mRNA expression of cardiomyocyte-specific cytoskeleton genes (*MYH6, MYH7, TNNT2, TNNI1, TNNI3*) in hPSC-CMs under 1 g and μg conditions. **q** The protein expression of TNNI1 and TNNI3. **r** The relative mRNA expression of cardiomyocyte-specific calcium handling genes (*RYR2, ATP2A2, SLC8A1, CACNA1C*) in hPSC-CMs under 1 g and μg conditions. Data are presented as mean ± SEM; Student’s *t* test; *P < 0.05, **P < 0.01, ns not significant

For the aerospace biomedical research plan (Fig. 1a), two human pluripotent stem cell (hPSC) lines were prepared: human induced pluripotent stem cells (hiPSCs) and H9 human embryonic stem cells with GCaMP6f knock-in (GCaMP). The hiPSC line was generated by reprogramming urine renal epithelial cells from a healthy adult volunteer (Supplementary Fig. 1a). The GCaMP cell line was developed to illustrate calcium signaling visually.^22,23^ Both hiPSCs and GCaMP cells expressed the classical stemness makers OCT4 and SOX2 (Supplementary Fig. 1b, c). The hiPSCs were further verified by forming embryoid bodies and teratoma with three germ layers (Supplementary Fig. 1d, e), and karyotype analysis showed no chromosome abnormality (Supplementary Fig. 1f).

The generated hiPSCs and GCaMP cells were then differentiated into beating cardiomyocytes by modulating WNT signaling in the CDM3 medium. Both hiPSC-CMs and GCaMP cell-derived cardiomyocytes (GCaMP-CMs) exhibited spontaneous beating and high expression of cardiac markers α-Actinin and cTnT (Supplementary Fig. 1g, h). Highly purified cardiomyocytes were obtained (Supplementary Fig. 1i, j). In particular, circularly permuted green fluorescent calcium signals were detected in GCaMP-CMs, which were synchronized with cell contraction. These fluorescence signals allowed monitoring the calcium dynamics and mechanical signals of cardiomyocytes on the CSS. After purification, hiPSC-CMs and GCaMP-CMs were seeded in space cell culture chambers, loaded into the cell culture apparatus equipped with an automated fluid exchange system (Fig. 1b), and launched to the Tianhe core module of the CSS. The cardiomyocytes aboard the CSS were exposed to the microgravity condition (μg) or the simulated ground environment (1 g) for 6 days in culture condition, followed by sample collection.

### Space microgravity-induced adaptations in hPSC-CMs

As space microgravity may reduce the heart rate in astronauts,^24,25^ we first tested the contractile properties of hPSC-CMs in microgravity. Compared to the 1 g group on the CSS, the cell size of cardiomyocytes showed a downward trend in the μg group. At the same time, no significant difference in mean fluorescence intensity (MFI) for peak calcium signal was detected between the two groups (Fig. 1c–e). Compared with the simulated gravity group shown in Supplementary Movie 1, space microgravity exposure obviously reduced the beating frequency of GCaMP-CMs (Fig. 1f and Supplementary Movie 2).

Contractility is directly related to the intracellular calcium concentration in the cardiomyocytes. Taking advantage of the GCaMP calcium sensor, we further assessed the Ca^2+^-handling properties in GCaMP-CMs (Fig. 1g). There was no significant difference in parameters of calcium transient amplitude and time to peak between the 1 g group and μg group (Fig. 1h, i). However, cardiomyocytes exposed to microgravity exhibited a significant increase in decay time, indicating a decreased calcium recycling rate (Fig. 1j). The abnormal intracellular calcium cycling may directly cause a reduced beating rate. We also analyzed the changes in tau function, and the data showed a remarkable increase under microgravity condition, which reflects an impaired function in relaxation (Fig. 1k).

The sarcomeres represent the functional units of cardiomyocytes that directly participate in cell contraction and remodeling. We attended to determine the effects of microgravity on sarcomere organization. Immunofluorescence staining for sarcomere protein cTnT and α-Actinin illustrated a relatively unstandardized structural arrangement with less strained and unordered sarcomeres in cardiomyocytes after exposure to space microgravity (Fig. 1l, m). Compared to the 1 g group, cardiomyocytes in the μg group have a less organized sarcomere structure, demonstrating reduced sarcomere length (1.99 μm ± 1.11 vs 1.45 μm ± 0.87) and cTnT content (Fig. 1l–o). Furthermore, we also found changes in sarcomere gene expressions, including TNNI1 and TNNI3,^26^ indicating a disordered cytoskeleton structure under a microgravity environment (Fig. 1p, q). The sarcoplasmic/endoplasmic reticulum calcium ATPase (SERCA) pump could bring cytosolic Ca^2+^ into the sarcoplasmic reticulum to initiate muscle relaxation and maintain low intracellular calcium.^27^ The L-type Ca^2+^ channel (LTCC) has been proven to be a target for regulating the beating rate.^28,29^ Consistent with the change of calcium cycling, we detected an abnormal expression of ATP2A2 and calcium voltage-gated channel subunit alpha1 C (CACNA1C) in microgravity-exposed cardiomyocytes (Fig. 1r). In all, space microgravity can lead to sarcomere rearrangement and abnormal expression of calcium cycling-related genes in hPSC-CMs and consequently induce altered contractility of cardiomyocytes.

### Altered thiamine utilization in space microgravity-exposed hPSC-CMs

**Fig. 2:**
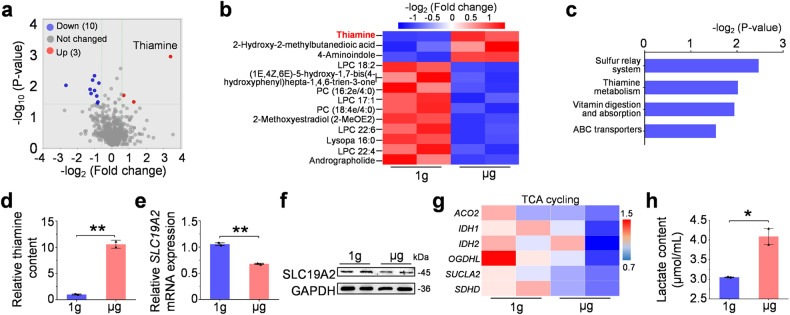
Metabonomic analysis of cell supernatants under space microgravity condition. **a** The volcano plot showing differential metabolites of cell supernatant under 1 g and μg conditions. **b** The heatmap plot showing that 3 metabolites were higher in μg cell supernatant, and 10 metabolites were higher in 1 g cell supernatant. **c** The KEGG analysis of differential metabolites. **d** Thiamine content in the cell culture supernatant of hPSC-CMs under 1 g and μg conditions. **e** The relative mRNA expression of SLC19A2 (thiamine transporter) in hPSC-CMs under 1 g and μg conditions. **f** The protein expression of SLC19A2 in hPSC-CMs under 1 g and μg conditions. **g** Transcriptomic analysis showing decreased expression profile of genes related to TCA cycles in hPSC-CMs under μg conditions. **h** The comparison of lactate content in cell culture supernatant of hPSC-CMs under 1 g and μg conditions. Data are presented as mean ± SEM; Student’s *t* test; *P < 0.05, **P < 0.01

Cardiomyocytes are high-energy demanding muscle cells as they require energy to fuel contraction. To determine whether changes in metabolism accompanied the altered contractility of cardiomyocytes under microgravity condition, we assessed the metabolite content in cell culture supernatant by metabolomic analysis. We identified 824 metabolites, including 525 positive and 299 negative iron mode metabolites (Fig. 2a). Of these, 13 metabolites showed a significant difference between the 1 g and μg groups (Fig. 2b and Supplementary Table 3). These differential metabolites were primarily involved in the sulfur relay system, thiamine metabolism, vitamin digestion & absorption, and ABC transporters (Fig. 2c). We further performed transcriptomic analysis and found enrichments of differentially-expressed genes in lipid metabolism, metabolism of cofactors and vitamins, and carbohydrate metabolism in terms of metabolism catalog (Supplementary Fig. 2a–c and Supplementary Table 4). The above results indicate that significant metabolism changes, particularly energy metabolism, occur in space microgravity.

Thiamine (vitamin B1) is an essential water-soluble vitamin that an organism cannot synthesize.^30^ Interestingly, we noticed that thiamine was significantly enriched in the cell culture supernatant of microgravity-exposed hPSC-CMs compared with the 1 g group (Fig. 2d), indicating that hPSC-CMs cannot uptake and utilize thiamine normally. Consistently, microgravity-exposed hPSC-CMs showed low mRNA and protein expression of thiamine transporter SLC19A2 (Fig. 2e, f). In contrast, the other two members, SLC19A1 and SLC19A3, had no significant change (Supplementary Fig. 2d, e). Thiamine is critical for pyruvate dehydrogenase and alpha-ketoglutarate dehydrogenase activity in the tricarboxylic acid (TCA) cycle. The transcriptomic data consistently indicated that space microgravity could inhibit the essential gene expression of pyruvate oxidation and the TCA cycle (Fig. 2g). Consequently, lactic acid usually converted from excessive pyruvate, was significantly increased in the extracellular medium upon microgravity exposure (Fig. 2h). We, therefore, speculated that cardiomyocytes could adapt to the microgravity environment by metabolic reprogramming, in which thiamine might play a critical role.

### Thiamine antagonist leads to structural and functional disorders in hPSC-CMs

**Fig. 3:**
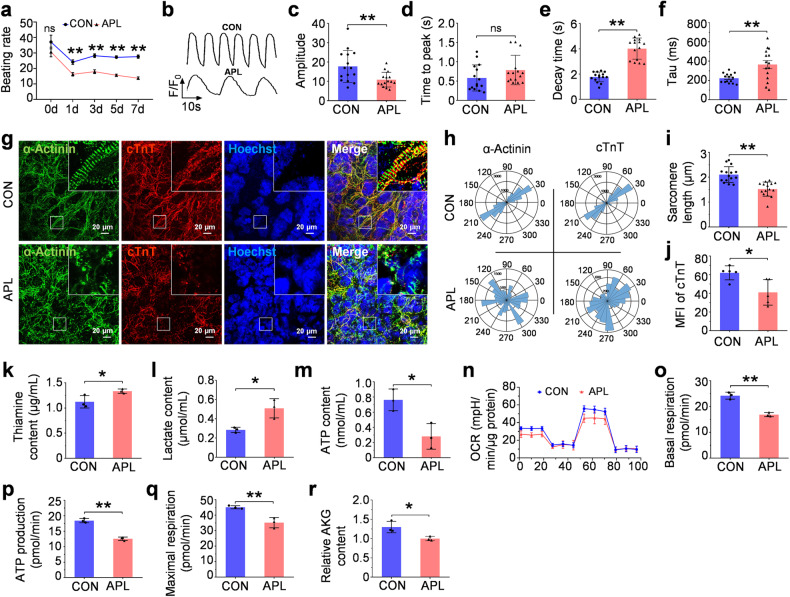
Thiamine antagonist disturbs cardiomyocytes. **a** The beating rate analyses of GCaMP-CMs in the control group (CON) and thiamine antagonist group (amprolium, APL). **b** Representative calcium transients of GCaMP-CMs treated with CON and APL. **c**–**e** The quantitative analyses of amplitude (**c**), time to peak (**d**), decay time (**e**), and tau (**f**) of GCaMP-CMs treated with CON and APL. **g** Immunofluorescence staining of cytoskeleton proteins in GCaMP-CMs (α-Actinin, green; cTnT, red; Hoechst 33342, blue). **h** Representative polar histogram of the myofibrillar alignment in GCaMP-CMs. **i**, **j** The quantitative analyses on sarcomere length (**i**) and cTnT content (**j**) of GCaMP-CMs treated with CON and APL. **k** The thiamine content of cell culture supernatant of GCaMP-CMs. **l** The comparison of lactate content in the cell culture supernatant of GCaMP-CMs treated with CON and APL. **m** The quantitative analyses of ATP content in cell lysates of GCaMP-CMs treated with CON and APL. **n** Representative OCR traces of hiPSC-CMs treated with CON and APL were obtained using a Seahorse XFe24 Analyzer. Quantification of basal respiration (**o**), ATP production (**p**), and maximal respiration (**q**). **r** The α-ketoglutaric acid (AKG) content analysis under μg environment. Data are presented as mean ± SEM; Student’s *t* test or two-way repeated-measures ANOVA; *P < 0.05, **P < 0.01, ns not significant

We induced thiamine deficiency in GCaMP-CMs to validate this hypothesis by treatment with the thiamine analog—amprolium. Amprolium (APL) acts as a thiamine antagonist, blocking vitamin transport across cells.^31^ Similar phenotypes, including degressive beating rate (Fig. 3a), prolonged decay time, and tau (Fig. 3b–f), were presented after thiamine deprivation in GCaMP-CMs. Furthermore, APL treatment could lead to apparent cytoskeleton reorganization, as evidenced by the disorder sarcomere structure, shortened sarcomere length, and reduced cTnT expression (Fig. 3g–j). We also found impaired utilization of thiamine, by APL treatment significantly increased the contents of residual thiamine and exported lactate in the cell culture supernatant (Fig. 3k, l). It decreased the ATP synthesis (Fig. 3m), which could further decline the beating rate of CMs.

The Seahorse metabolic flux assay also demonstrated that oxidative phosphorylation, including basal respiration, ATP production, and maximal respiration, decreased after APL treatment (Fig. 3n–q). The decline of α-ketoglutaric acid, the product of the TCA cycle, indicated that APL could slow the TCA flux and ATP production (Fig. 3r). Taken together, the decreased utilization of thiamine induces TCA cycle disruption, which may account for less energy expenditure for cell contraction and cytoskeletal assembly in microgravity-exposed cardiomyocytes.

### Thiamine supplementation antagonized the effect of microgravity on hPSC-CMs

**Fig. 4:**
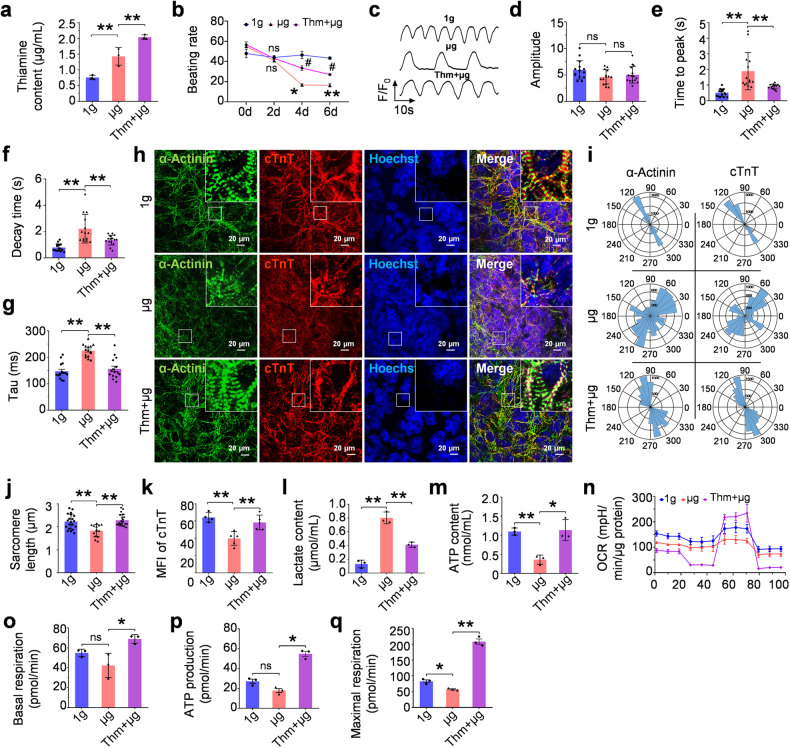
Thiamine supplementation relieves microgravity-induced cardiac adaption. **a** The thiamine content of cell culture supernatant of GCaMP-CMs under 1 g, simulated microgravity (μg) conditions. Partial microgravity-stimulated GCaMP-CMs were synchronously treated with thiamine (Thm+μg). **b** The beating rate analyses of GCaMP-CMs in 1 g, μg, and Thm+μg groups. **c** Representative calcium transients of GCaMP-CMs under 1 g, μg, and Thm+μg conditions. **d**–**g** The quantitative analyses of amplitude (**d**), time to peak (**e**), decay time (**f**), and tau (**g**) of GCaMP-CMs. **h** Immunofluorescence staining of cytoskeleton proteins in GCaMP-CMs under 1 g, μg, and Thm+μg conditions (α-Actinin, green; cTnT, red; Hoechst 33342, blue). **i** Representative polar histogram of myofibrillar alignment in GCaMP-CMs under 1 g, μg, and Thm+μg conditions. **j**, **k** The quantitative analyses on sarcomere length (**j**) and cTnT content (**k**) of GCaMP-CMs under 1 g, μg, and Thm+μg conditions. **l** The analyses of lactate content in cell supernatant of GCaMP-CMs under 1 g, μg, and Thm+μg conditions. **m** The quantitative analyses of ATP content in cell lysates of GCaMP-CMs. **n** Representative OCR traces of hiPSC-CMs in 1 g, μg, and Thm+μg groups were obtained using a Seahorse XFe24 Analyzer. Quantification of basal respiration (**o**), ATP production (**p**) and maximal respiration (**q**). Data are presented as mean ± SEM; one-way ANOVA or two-way repeated-measures ANOVA; * or ^#^*P* < 0.05, **P < 0.01, ns not significant

The above evidence prompted us to determine whether thiamine supplementation could prevent microgravity-induced changes in calcium dynamics and contractility in CMs. GCaMP-CMs were exposed to simulated microgravity using a rotary cell culture system (RCCS) for 6 days, with or without a thiamine supplement in the culture medium. The experimental results showed that cellular response to simulated microgravity on thiamine utilization was similar to that of space microgravity (Fig. 4a). Thiamine supplementation led to apparent resistance to microgravity-induced attenuation of the beating rate and Ca^2+^ handling capability (Fig. 4b–g). Furthermore, thiamine supplementation could protect CMs against microgravity-induced cytoskeletal remodeling and depolymerization (Fig. 4h–k). Meanwhile, the lactic acid content in the cell culture supernatant was significantly reduced by thiamine supplementation, accompanied by increased ATP in CMs treated with thiamine (Fig. 4l, m). The Seahorse metabolic flux assay showed that thiamine supplementation could rescue the slow TCA flux caused by simulated microgravity (Fig. 4n–q).

For further gain-of-function study, a lentiviral vector was constructed and transfected to induce the expression of SLC19A2, also known as the thiamine transporter, in hiPSC-CMs in vitro (Supplementary Fig. 3a). SLC19A2 overexpression (μg + OE) could reverse the adaptive changes of CMs in response to microgravity. Thiamine content in cell supernatant dropped significantly, suggesting that thiamine intake improved after overexpression of SLC19A2 (Supplementary Fig. 3b). It could partially alleviate the disorder sarcomere structure and reverse the downward trend in the beating rate of cardiomyocytes (Supplementary Fig. 3c, d). Furthermore, overexpression of SLC19A2 was able to ameliorate abnormal metabolism, increased lactate, and oxidative phosphorylation defects caused by simulated microgravity (Supplementary Fig. 3e–i). Taken together, the above data indicate that thiamine supplementation plays an essential role in protection against microgravity-induced phenotypic changes of CMs by enhancing the TCA cycle.

### Thiamine treatment alleviated simulated microgravity-induced mouse cardiac dysfunction

**Fig. 5:**
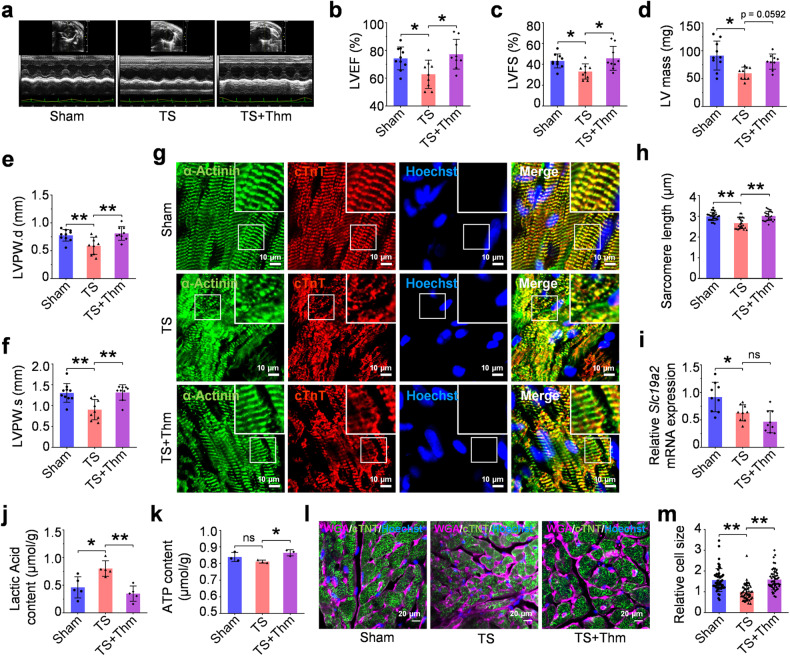
Thiamine treatment attenuates simulated microgravity-induced mouse cardiac dysfunction. **a** Representative M-mode echocardiographic images obtained from Sham, tail suspension (TS), and tail suspension + thiamine (TS+Thm) mice on day 28. **b**–**f** Assessment of echocardiographic parameters from the mice in Sham, TS, and TS+Thm groups. LVEF: ejection fraction, LVFS: fractional shortening, LV mass: left ventricle mass, LVPW.d: left ventricular posterior wall thickness at end-diastole, LVPW.s: Left ventricular posterior wall thickness at end systolic. **g** Immunofluorescence staining of cytoskeleton proteins (α-Actinin, green; cTnT, red; Hoechst 33342, blue). **h** The quantitative analyses on sarcomere length in three groups. **i** The quantitative analyses of SLC19A2 in heart tissue lysate. **j**, **k** The quantitative analyses of ATP production and lactic acid content in heart tissue lysate. **l**, **m** The cell size analysis by WGA staining in Sham, TS, and TS+Thm groups. Data are presented as mean ± SEM; one-way ANOVA; *P < 0.05, **P < 0.01, ns not significant

We then assessed whether thiamine supplementation could reverse simulated microgravity-induced cardiac functional changes in vivo. The tail-suspension (TS) animal model with 30° head-down tilt for 28 days in mice was used to redistribute body fluid, which has been proven an effective method to simulate microgravity.^32,33^ The mice simultaneously received daily oral gavage with thiamine or water. According to the echocardiographic data, tail suspension altered cardiac function in mice, as evidenced by the significant reduction of LVEF and LVFS in mice compared to that in control (Fig. 5a–c). Meanwhile, the LV mass, LVPW.d, and LVPW.s were significantly decreased in TS mice, indicating cardiac atrophy in these mice (Fig. 5d–f). Furthermore, thiamine treatment could remarkably reverse the atrophic phenotype and improve cardiac systolic and diastolic function (Fig. 5a–f).

In addition, cytoskeletal remodeling and depolymerization were found, and the expression of SLC19A2 was significantly reduced in the TS group (Fig. 5g–i). Thiamine supplementation could protect the myocardium against the above changes, although it failed to restore the SLC19A2 expression. We further detected the content of lactic acid and ATP in heart tissue. Thiamine supplementation could significantly improve ATP production and decrease the lactic acid content in TS mice (Fig. 5j, k). The WGA staining demonstrated that the cell size of cardiomyocytes showed a downward trend in TS mice, while thiamine supplementation could reverse these changes (Fig. 5l, m). The above data indicate that thiamine supplementation could protect the heart from TS-induced structural and functional alterations.

## Discussion

**Fig. 6:**
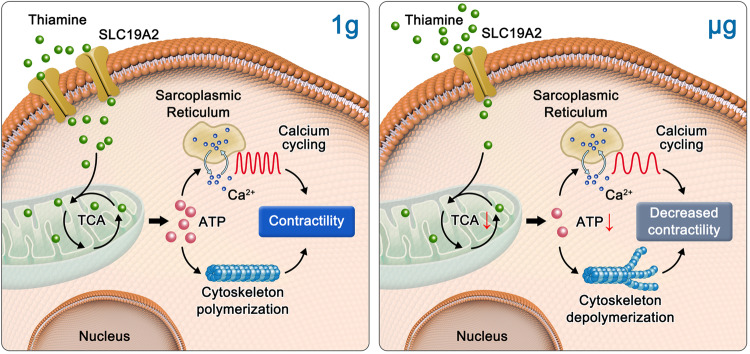
Impaired utilization of thiamine is responsible for microgravity-induced adaptive changes in cardiomyocytes and cardiac function

In this study, we conducted the first astrobiological study on the China Space Station. We proposed a whole new viewpoint for space microgravity-induced adaptation in the heart by virtue of hPSC-CMs. Microgravity exposure reduces the intake and utilization of thiamine in human cardiomyocytes, further disrupting the TCA cycle and reducing ATP production. The shortage of ATP production would lead to cytoskeletal remodeling and calcium cycling prolongation, which weakens cardiomyocyte contraction (Fig. 6). The exogenous supplementation of thiamine could relieve simulated microgravity-induced structural, metabolic, and functional alterations in human cardiomyocytes and improve cardiac function in mice under tail-suspension conditions. This study provides new insights into weightlessness physiology and intervention strategies.

Decreased contractility and cardiac atrophy caused by microgravity have been reported in previous studies.^5,24,25^ During spaceflight, we recaptured these phenotypes at the cellular levels, as indicated by a prolonged calcium recycling, decreased beating rate, and less structured cytoskeleton in hPSC-CMs, which is consistent with a recent study conducted in the International Space Station.^11,16^ This study found that hPSC-CMs presented a slower TCA flux and less ATP production after microgravity exposure. In addition, metabolomic and transcriptomic data also revealed a series of energy metabolism pathways that have been changed in microgravity condition, such as lipid, vitamin, and carbohydrate metabolism. In brief, these adaptive changes caused by microgravity are the basis of pathology-like changes, such as structural and metabolic remodeling.

Mitochondria stress is regarded as a central biological hub and is critical in adaptative changes during spaceflight conducted by the NASA Twin Study and others.^9,34,35^ Interestingly, a recent study demonstrated that mitochondria-mediated pathways were still enriched in cardiomyocytes even when they had returned to normal gravity for 10 days, indicating the incomplete restoration of normal mitochondrial function.^16^ The TCA cycle is one of the most important ways to generate ATP in mitochondria.^36^ In this study, TCA cycle-related gene expression decreased in the microgravity group, indicating a slowdown in metabolic flux. This further reduced ATP production to adapt to the low-energy requirement under microgravity condition. Thus, the mitochondrial TCA cycle may serve as a target against microgravity stress during human space travel or promote heart recovery after returning to earth. Mitochondria stress signaling is also relative to glutathione synthesis and de novo synthesis of amino acids such as aspartate and proline.^37–39^ We checked the metabolomics data, and there is no significant difference in the level of glutathione and proline between 1 g and μg groups. Transcriptomics data also demonstrated that the de novo synthesis of glutathione, aspartate, and proline seem to be independent of gravity since the key enzymes responsible for their synthesis showed no significant difference between 1 g and μg groups. However, it cannot be excluded due to limited experimental data, and more attention will be paid to the de novo synthesis of amino acids under space microgravity in the future.

Several rate-limiting enzymes may act as critical regulatory targets in TCA cycles, including citrate synthase, isocitrate dehydrogenase, α-ketoglutarate dehydrogenase, and pyruvate dehydrogenase. Thiamine, acting as a coenzyme for α-ketoglutarate dehydrogenase and pyruvate dehydrogenase, is a crucial regulator of the TCA cycle.^40^ In previous studies, thiamine deficiency has been suggested to be associated with many cardiovascular diseases, which may be related to the decline in energy demand.^18,30^ Here, we found that thiamine intake in cardiomyocytes was impaired in microgravity, along with the down-regulation of thiamine transporter SLC19A2. Strikingly, after thiamine blockage by its antagonist, hPSC-CMs exhibited an apparent decline in mitochondrial ATP generation and weakened contraction. Because SCL19A2 did not change dramatically (~50%), we also detected the other two members, SLC19A1 and SLC19A3. However, we did not find significant differences. These data indicate that either SCL19A2 plays a master role in transporting thiamine or other unidentified transporters might coordinate with SCL19A2 in microgravity condition.

Some countermeasures have been assessed to overcome cardiovascular adaptations during and after long-duration spaceflight, including penguin suit, exercise, and nutrition.^41,42^ A recent study suggested that cardiac mass seemed to be preserved after spaceflight on the ISS, which benefited from an intense exercise program (>2 h daily).^43^ Despite all this, these countermeasures may be imperfect and insufficient during long-duration spaceflight (>12 months). Nutritional interventions have proven effective in maintaining or restoring other organ functions such as protein or amino acids, cofactors (vitamins), and inorganic salts.^44,45^ The B vitamins, which have been reported as predictors of ophthalmic abnormalities and low status, may lead to endothelial dysfunction during and after flight.^46^ Meanwhile, thiamine deficiency may lead to congestive heart failure, and thiamine treatment could preserve ATP levels and maintain contractility performance.^18,47–49^ Our study indicated that thiamine supplementation could rescue microgravity-induced cardiomyocyte adaptive changes and benefit functional recovery in both human cardiomyocytes and animals. We believed thiamine supplementation might be a potential strategy to attenuate excessive adaptation to microgravity in long-term spaceflight and promote functional recovery post-flight. In the following study, we would like to test whether thiamine supplements could exert protective effects on astronauts.

Most previous studies in cardiac responses to microgravity were conducted either in non-human models or under simulated weightless circumstances, which may not perfectly capture the cellular reactions and physiological processes influenced by real microgravity.^50^ Taking full advantage of the cell maintenance and imaging system on the CSS, our study is the first to explore the dynamic responses of human cardiomyocytes to microgravity on the CSS and the first to monitor the dynamic calcium transient signals in hPSC-CMs during a spaceflight. Besides, we introduced a 1 g control group at CSS to eliminate the impact of other environmental factors, such as hyper-gravity during the launching process, cosmic radiation, and circadian rhythm changes during spaceflight. However, it will be more scientific and reasonable to set a 1 g control group on Earth after solving the problem of environmental differences between low-earth orbit and Earth’s surface.

According to the literature, the cardiomyocytes derived from pluripotent stem cells were heterogeneous, including ventricular-, atrial- and nodal-like cardiomyocytes. Despite this, the ventricular myocytes are considered as the largest proportion and play a decisive role in response to microgravity.^51^ In addition, our study was based on 2D-cultured hPSC-CMs, which typically exhibited fetal-like characteristics and remained immature in a dish.^52^ Despite complex challenges, fully matured hPSC-CMs and 3D cardiac models, such as cardiac organoids and engineered cardiac tissues, are needed to better model the human heart’s microgravity-induced structural and functional changes in vitro. In the in vivo study, the tail-suspension model, which is still commonly used to study the responses of physiological systems to spaceflight, was introduced to verify the performance of thiamine. However, some potential differences on stress responses between between tail-suspension model and space microgravity may affect the reliability and accuracy of the results. Upon establishing the fundamental experimental conditions on the CSS, further validation experiments based on mammalian animals will become possible.

In conclusion, we found that hPSC-CMs experienced adaptive changes and maintained low-energy homeostasis in spaceflight. The cardiomyocytes acclimatized themselves to microgravity by reducing TCA flux and ATP production. As cofactors of crucial enzymes during TCA cycling, thiamine utilization was hindered when exposed to microgravity. Forced blockage of thiamine intake can reduce the overall phenotypes of microgravity exposure. Thiamine supplementation may be a potential strategy to mitigate adaptive changes and prevent pathological remodeling due to long-term spaceflight. This study may provide a new perspective on space medicine and pave the way for further investigation.

### Limitation of this study

Considering the differences between space microgravity and simulated microgravity, further detailed investigation into thiamine-induced cardiac response to microgravity are necessary to be verified in space stations. After long-duration spaceflight, the adaptive changes may turn into pathological changes in the cardiovascular system.^53,54^ Due to space resource limitations, in this study, we only could collect the data from short-term spaceflight which also serve as a valuable reference for further long-term study. In addition, the hiPSC-CMs used in this study were single-cell type and relatively immature, which may not fully recapture the physiological characteristics of the adult heart.^55,56^ Using mammalian animals or human multicellular tissue-like structures, such as cardiac organoids, engineered heart tissues, and organ chips, will provide a more advanced model and allow the superior study of interactions among different cell types in the heart. Conclusively, the multidimensional platforms and long-time study design may sufficiently illustrate microgravity-induced cardiac response and successfully develop countermeasures and treatment strategies against space microgravity.

## Materials and methods

### Cell culture apparatus in the Tianhe core module of China Space Station

The cell culture apparatus was designed and engineered to match the specifications and safety standards of the Tianhe core module of China Space Station (CSS). It contains three essential parts: cell culture units, centrifuge, and dual-magnification microscope. Each cell culture unit includes a cell culture set, storage bags for cell culture medium, digestive solution, fixative solution and discard solution, micro peristaltic pumps for liquid flow, and an optical source. All the cell culture units were placed in a conventional cell culture condition (37 °C and 5% CO_2_), with some exposed to microgravity (μg) directly and some put in a horizontal centrifuge to simulate the gravity on the earth (1 g). The dual-magnification microscope (×10 and ×20) was equipped to capture bright-filed images automatically according to the uploaded commands. Besides the automatic microscope, another microscope is equipped to observe cells in bright-field and fluorescence modes manipulated by astronauts.

### Generation of human induced pluripotent stem cells and GCaMP cell line

As previously published, human induced pluripotent stem cells (hiPSCs) were reprogrammed from renal epithelial cells.^57^ Experiments with donated urine samples were approved by the Ethics Committee of Soochow University, and the informed consent was signed by all subjects. The urine sample was collected according to a published protocol with slight modifications,^58^ and then the isolated renal epithelial cells were maintained in the REBM urine cell medium (LONZA, Switzerland). Renal epithelial cells lower than passage five were used for somatic reprogramming by lentiviral transfection of Yamanaka factors. After the colonies appeared, they were mechanically picked onto Matrigel (Corning, USA) coated plates and routinely maintained in mTeSR^TM^1 medium (Stemcell, Canada) at 37 °C with 5% (v/v) CO_2_. The generated hiPSCs were further identified by stemness marker detection, teratoma formation, embryonic body formation, and karyotype analysis.

The GCaMP human embryo stem cell line was generated to illustrate the calcium signaling visually. GCaMP6f gene was introduced into H9 ESCs by the CRISPR technique. After screening and identification, the genome-edited cell line, named GCaMP, has been routinely used to study calcium signaling in cardiomyocytes.^22,23^

### Cardiomyocyte differentiation

When the confluence reached ~90%, hPSCs were initiated to differentiate into cardiomyocytes as previously described.^59^ Briefly, cells were successively cultured in CDM3 differentiation medium containing 4 μM CHIR99021 (Sigma-Aldrich, USA) for 2 days and 2 μM C59 (Selleck Chemicals, USA) for 2 days and then were maintained in the CDM3 differentiation medium. The medium was refreshed daily, and spontaneous beating appeared routinely from days 9–10. The differentiated cells were further purified by culturing in glucose-free RPMI 1640 (Thermo Fisher, USA) supplemented with 5 mM sodium DL-lactate (Sigma-Aldrich, USA) for 3 days. Purified cardiomyocytes (1 × 10^6^) were plated onto 0.1% gelatin-coated space cell culture chambers in CDM3 medium.

### Cardiomyocyte cultivation on the China Space Station

Human PSC-derived cardiomyocytes were transported into the CSS during the China crewed spaceship SZ-13 spaceflight mission, which was launched by the Long March 2 F carrier rocket at the Jiuquan Satellite Launch Center on October 16, 2021. Twenty hours before launch, cells were observed and imaged as the initial states of cells on the ground. After the cells were transferred into the Tianhe core module of the CSS by SZ-13 manned cargo spacecraft, some cell culture chambers were assembled into cell culture units for microgravity exposure, and others for exposure to stimulated 1 g gravity. The cell culture medium was changed every 36 h. During the 144 h on-orbit experiment, images and videos in space were taken automatically every 24 h and manually operated by astronauts at three time points: 15–24 h, 75–99 h, and 130–142 h. For GCaMP-derived cardiomyocytes, the fluorescent mode was switched to record calcium signaling. At the end of the experiment, the cell culture medium was collected for biochemical analysis, and cells were collected for RNA and histochemical studies. All the samples were stored for the remaining flight and returned to the Earth with the SZ-13 manned spacecraft re-entry capsule on April 16, 2022.

### Cell cultivation with supplementation of thiamine and its antagonist

Microgravity condition was simulated using a rotary cell culture system (RCCS) platform. To explore the biological effects of thiamine on cardiomyocytes, we seeded GCaMP-CMs in 12.5-cm^2^ culture flasks and filled them with a complete medium supplemented with/without 1 mM of amprolium-HCl (thiamine analog) (Selleck, USA) or 10 μM thiamine-HCl (Selleck, USA). Amprolium (APL) acts as a thiamine antagonist, blocking the transport of vitamin B1 across the cell member.^31^ These culture flasks were fixed on the RCCS and rotated at a speed of 10 rpm for 6 consecutive days, equivalent to a 0.001× *g* microgravity environment. The control group was maintained in the same culture condition except for gravity exposure. After that, all the cardiomyocytes and their supernatant were collected for further experiments.

### RNA extraction and RNA sequencing

Total RNA extracted from the cardiomyocytes or heart tissue was used in downstream applications, including real-time PCR and RNA sequencing (RNA-Seq). After the cells were lysed with TRIzol^TM^ Reagent (Thermo Fisher, USA), the total RNA samples were subjected to the library preparation and sequenced on the Illumina HiSeq platform (Illumina, USA) by Novogene (China). Transcript abundances in each sample were calculated and scaled to FPKM (fragments per kilobase of transcript per million mapped reads). The differential expression analysis of the two groups was performed using the R package, and the *P* values were adjusted using the Benjamini & Hochberg method. Standard RNA-Seq analysis was executed as previously described.^60^ All primers are listed in Supplementary Table 1.

### Metabolome analysis

Cell supernatant from different groups was analyzed using a Q ExactiveTM Plus orbitrap (Thermo Fisher, USA) that consisted of a 1290 LC system, a Jetstream electrospray ionization source, and a 6540 UHD accurate-mass qTOF spectrometry. Reversed-phase and hydrophilic interaction chromatography were adopted. Data in both positive and negative polarity were acquired. For potential biomarker selection, variable importance in projection >1 and fold change >1.5 or <0.67 were set for metabolites with significant differences.

### Immunofluorescence staining

After returning to the earth, the fixed cardiomyocytes were adopted for immunofluorescence staining against α-Actinin (Abcam, USA) and cTnT (Proteintech, China) to illustrate the cytoskeleton morphology, and the nuclei were stained with Hoechst 33342 (Molecular Probes, USA). The labeled cells were imaged by confocal microscopy (ZEISS, Germany). ImageJ software analyzed the sarcomere length by calculating the distance between intensity peaks. Five sarcomeres were measured along the long axis direction and then averaged to measure sarcomere length. A large number of sarcomeres (*n* > 10) were calculated to compare the sarcomere length.^22^ The ImageJ and Matlab software were used for orientation analysis of the sarcomere structure and myofilament arrangement. A higher-order cytoskeleton presents a highly centralized orientation, and any cytoskeletal remodeling and depolymerization will lead to a random orientation. To quantify the level of cTnT, we acquired a minimum of five images to analyze fluorescent intensity. In addition, we measured the cell size of cardiomyocytes after Alexa Fluor 647-WGA staining (Thermo Fisher, USA) to assess whether cardiac atrophy occurred in tail-suspension mice. All antibodies are listed in Supplementary Table 2.

### Detection for thiamine, lactic acid, α-ketoglutarate and ATP

The content or levels of thiamine, lactic acid, α-ketoglutarate (AKG), and ATP were detected by commercially available kits (Solarbio, China) according to the instructions, respectively.

### Seahorse assay

The Seahorse XFe24 Analyzer was utilized to measure the oxygen consumption rates (OCR) in hPSC-derived cardiomyocytes as previously described.^22^ Generally, the cardiomyocytes were seeded in gelatin-coated Seahorse XF culture microplates, and the OCR was measured after successive additions of oligomycin (2 μg/mL), FCCP (1 μM), and rotenone (0.5 μM) plus antimycin A (0.5 μM). The OCR values were then normalized to the total protein concentration per well.

### Western blot

Western blot analysis was conducted following the previously described protocol.^57^ Antibodies against SLC19A2 (BBI, China), TNNI1 (Proteintech, China), and TNNI3 (Proteintech, China) were employed for protein detection. Quantitative analysis was carried out using ImageJ software. All antibodies were listed in supplementary Table 2.

### Lentiviral production and transduction

The overexpression of SLC19A2 was achieved by using lentivirus vectors harboring the full-length SLC19A2 gene, which was designed and synthesized by Ribobio (China). Lentivirus vectors were transfected into 293 T cells, along with the packaging plasmids, using lipofectamine 2000 (Thermo Fisher, USA). Virus particles in the cell culture supernatants were harvested every 24 h three times and further concentrated with PEG-8000 (Sigma-Aldrich, USA). Transduction media containing virus particles and polybrene (1 μg/μL, Polysciences, USA) was used to infect cardiomyocytes for further study.

### Animal experiments

Mice were maintained under a 12/12 h light/dark cycle at 23 ± 2 °C. All animal procedures were approved by the Laboratory Animal Research Committee of Soochow University. Briefly, adult mice (2 months old) were randomly assigned to three groups (*n* = 10 per group), including the Sham group (Sham), tail-suspension group (TS), and tail-suspension plus thiamine group (TS+Thm). The mice in the TS and TS+Thm groups were subjected to tail suspension, which involved maintaining a 30° head-down tilt for 28 days, as previously described.^32,33^ All mice were allowed ad libitum access to food and drinking water. Tail-suspension mice received daily gavage with thiamine (50 mg/kg) or water. At day 28, all mice underwent echocardiographic evaluation and were subsequently euthanized under anesthesia. The heart samples were collected for further analysis.

### Echocardiography

As previously described, echocardiography was performed using a VisualSonics Vevo2100 system equipped with a medium-frequency (30 MHz) MS-400 transducer.^61^ The mice were anesthetized with 2% inhalant isoflurane, and transthoracic echocardiographic analysis was carried out using a 12-MHz probe. M-mode images were analyzed to assess changes in various parameters, including left ventricular mass (LV mass), LV ejection fraction (LVEF), LV fraction of shortening (LVFS), LV end-systolic posterior wall thickness (LVPW.s), LV end-diastole posterior wall thickness (LVPW.d). The studies and analysis were conducted following a randomized, double-blind methodology.

### Data analysis

Comparisons between the two groups were performed using Student’s *t* test. Comparisons among multiple groups were conducted with one-way analysis of variance (ANOVA) or two-way repeated-measures ANOVA with the Bonferroni post hoc test. Statistical significance was denoted by a *P* < 0.05. All data were presented as the mean ± standard error of the mean (SEM).

### Supplementary information

Supplemental figures

Supplement Video 1

Supplement Video 2

## Data Availability

The transcriptome and metabolome data reported in this paper have been deposited to the China National Center for Bioinformation/Beijing Institute of Genomics, Chinese Academy of Sciences (https://ngdc.cncb.ac.cn/omix: accession no. OMIX005947 and no. OMIX005952). The data that support the findings in the paper are available from the corresponding author upon reasonable request. Some data may not be made available because of non-disclosure agreement.

## References

[1] Williams DR (2003). The biomedical challenges of space flight. Annu. Rev. Med..

[2] Afshinnekoo E (2020). Fundamental biological features of spaceflight: advancing the field to enable deep-space exploration. Cell.

[3] Hupfeld KE, McGregor HR, Reuter-Lorenz PA, Seidler RD (2021). Microgravity effects on the human brain and behavior: dysfunction and adaptive plasticity. Neurosci. Biobehav. Rev..

[4] Strollo F, Vernikos J (2021). Aging-like metabolic and adrenal changes in microgravity: state of the art in preparation for Mars. Neurosci. Biobehav. Rev..

[5] Hughson RL, Helm A, Durante M (2018). Heart in space: effect of the extraterrestrial environment on the cardiovascular system. Nat. Rev. Cardiol..

[6] Scott JM, Stoudemire J, Dolan L, Downs M (2022). Leveraging spaceflight to advance cardiovascular research on Earth. Cir. Res..

[7] Baran R (2021). The cardiovascular system in space: focus on in vivo and in vitro studies. Biomedicines.

[8] Sy MR, Keefe JA, Sutton JP, Wehrens XHT (2023). Cardiac function, structural, and electrical remodeling by microgravity exposure. Am. J. Physiol. Heart Circ. Physiol..

[9] Nguyen HP, Tran PH, Kim KS, Yang SG (2021). The effects of real and simulated microgravity on cellular mitochondrial function. NPJ Microgravity.

[10] Lei X, Zhang W, Zhang Y, Zhao L (2022). Editorial: The regulating mechanisms of development, growth, and metabolism: from ground to space. Front. Cell. Dev. Biol..

[11] Walls S (2020). Prolonged exposure to microgravity reduces cardiac contractility and initiates remodeling in Drosophila. Cell. Rep..

[12] Cherednichenko G (2004). NADH oxidase activity of rat cardiac sarcoplasmic reticulum regulates calcium-induced calcium release. Cir. Res..

[13] Doenst T, Nguyen TD, Abel ED (2013). Cardiac metabolism in heart failure: implications beyond ATP production. Cir. Res..

[14] Gibb AA, Hill BG (2018). Metabolic coordination of physiological and pathological cardiac remodeling. Cir. Res..

[15] Bertero E, Maack C (2018). Metabolic remodelling in heart failure. Nat. Rev. Cardiol..

[16] Wnorowski A (2019). Effects of spaceflight on human induced pluripotent stem cell-derived cardiomyocyte structure and function. Stem Cell Rep..

[17] Connor MK, Hood DA (1998). Effect of microgravity on the expression of mitochondrial enzymes in rat cardiac and skeletal muscles. J. Appl. Physiol..

[18] DiNicolantonio JJ, Liu J, O’Keefe JH (2018). Thiamine and cardiovascular disease: a literature review. Prog. Cardiovasc. Dis..

[19] Gioda CR (2009). Impaired cellular contractile function in thiamine-deficient rat cardiomyocytes. Eur. J. Heart Fail..

[20] Roman-Campos D (2009). Cardiac structural changes and electrical remodeling in a thiamine-deficiency model in rats. Life Sci..

[21] Katare RG (2010). Vitamin B1 analog benfotiamine prevents diabetes-induced diastolic dysfunction and heart failure through Akt/Pim-1-mediated survival pathway. Circ. Heart Fail..

[22] Miao S (2020). Retinoic acid promotes metabolic maturation of human embryonic stem cell-derived cardiomyocytes. Theranostics.

[23] Li X (2019). MLP-deficient human pluripotent stem cell derived cardiomyocytes develop hypertrophic cardiomyopathy and heart failure phenotypes due to abnormal calcium handling. Cell. Death Dis..

[24] Liu Z (2015). Alterations in the heart rate and activity rhythms of three orbital astronauts on a space mission. Life. Sci. Space Res..

[25] Otsuka K (2016). Long-term exposure to space’s microgravity alters the time structure of heart rate variability of astronauts. Heliyon.

[26] Miki K (2021). ERRgamma enhances cardiac maturation with T-tubule formation in human iPSC-derived cardiomyocytes. Nat. Commun..

[27] Braun JL, Geromella MS, Hamstra SI, Messner HN, Fajardo VA (2021). Characterizing SERCA function in murine skeletal muscles after 35-37 days of spaceflight. Int. J. Mol. Sci..

[28] Schwoerer AP (2013). Enhanced Ca(2)+ influx through cardiac L-type Ca(2)+ channels maintains the systolic Ca(2)+ transient in early cardiac atrophy induced by mechanical unloading. Pflug. Arch..

[29] Acharya A (2019). Parabolic, flight-induced, acute hypergravity and microgravity effects on the beating rate of human cardiomyocytes. Cells.

[30] Smith TJ (2021). Thiamine deficiency disorders: a clinical perspective. Ann. N. Y. Acad. Sci..

[31] Teran MDM, de Moreno de LeBlanc A, Savoy de Giori G, LeBlanc JG (2021). Thiamine-producing lactic acid bacteria and their potential use in the prevention of neurodegenerative diseases. Appl. Microbiol. Biotechnol..

[32] Wang X (2013). miR-214 targets ATF4 to inhibit bone formation. Nat. Med..

[33] Wang L (2020). Mechanical sensing protein PIEZO1 regulates bone homeostasis via osteoblast-osteoclast crosstalk. Nat. Commun..

[34] Acharya A (2022). Microgravity-induced stress mechanisms in human stem cell-derived cardiomyocytes. iScience.

[35] da Silveira WA (2020). Comprehensive multi-omics analysis reveals mitochondrial stress as a central biological hub for spaceflight impact. Cell.

[36] Martinez-Reyes I, Chandel NS (2020). Mitochondrial TCA cycle metabolites control physiology and disease. Nat. Commun..

[37] Zhu J (2021). Mitochondrial NADP(H) generation is essential for proline biosynthesis. Science.

[38] Ritterhoff J (2020). Metabolic remodeling promotes cardiac hypertrophy by directing glucose to aspartate biosynthesis. Cir. Res..

[39] D’Amico D, Sorrentino V, Auwerx J (2017). Cytosolic proteostasis networks of the mitochondrial stress response. Trends Biochem. Sci..

[40] Marrs C, Lonsdale D (2021). Hiding in plain sight: modern thiamine deficiency. Cells.

[41] Scott JM (2023). Effects of exercise countermeasures on multisystem function in long duration spaceflight astronauts. NPJ Microgravity.

[42] Chaloulakou S, Poulia KA, Karayiannis D (2022). Physiological alterations in relation to space flight: the role of nutrition. Nutrients.

[43] Khine HW (2018). Effects of prolonged spaceflight on atrial size, atrial electrophysiology, and risk of atrial fibrillation. Circ. Arrhythm. Electrophysiol..

[44] Gao R, Chilibeck PD (2020). Nutritional interventions during bed rest and spaceflight: prevention of muscle mass and strength loss, bone resorption, glucose intolerance, and cardiovascular problems. Nutr. Res..

[45] Costa F (2021). Spaceflight induced disorders: potential nutritional countermeasures. Front. Bioeng. Biotechnol..

[46] Smith SM, Zwart SR (2018). Spaceflight-related ocular changes: the potential role of genetics, and the potential of B vitamins as a countermeasure. Curr. Opin. Clin. Nutr. Metab. Care.

[47] Witte KK (2005). The effect of micronutrient supplementation on quality-of-life and left ventricular function in elderly patients with chronic heart failure. Eur. Heart J..

[48] Vammen L (2023). Thiamine for the treatment of cardiac arrest-induced neurological injury: a randomized, blinded, placebo-controlled experimental study. J. Am. Heart Assoc..

[49] Yamada Y (2021). Thiamine treatment preserves cardiac function against ischemia injury via maintaining mitochondrial size and ATP levels. J. Appl. Physiol..

[50] Nishimura Y (2023). Technology using simulated microgravity. Regen. Ther..

[51] Funakoshi S (2021). Generation of mature compact ventricular cardiomyocytes from human pluripotent stem cells. Nat. Commun..

[52] Karbassi E (2020). Cardiomyocyte maturation: advances in knowledge and implications for regenerative medicine. Nat. Rev. Cardiol..

[53] Gallo C, Ridolfi L, Scarsoglio S (2020). Cardiovascular deconditioning during long-term spaceflight through multiscale modeling. NPJ Microgravity.

[54] Loktev SS, Ogneva IV (2019). DNA methylation of mouse testes, cardiac and lung tissue during long-term microgravity simulation. Sci. Rep..

[55] Kostina A, Volmert B, Aguirre A (2024). Human heart organoids: current applications and future perspectives. Eur. Heart J..

[56] Zushin PH, Mukherjee S, Wu JC (2023). FDA modernization Act 2.0: transitioning beyond animal models with human cells, organoids, and AI/ML-based approaches. J. Clin. Investig..

[57] Ye L (2022). Patient-specific iPSC-derived cardiomyocytes reveal abnormal regulation of FGF16 in a familial atrial septal defect. Cardiovasc Res..

[58] Yu M, Han X, Ye L, Lei W, Hu S (2022). Generation of human induced pluripotent stem cells from renal epithelial cells. Methods Mol. Biol..

[59] Yang J (2021). Intermittent starvation promotes maturation of human embryonic stem cell-derived cardiomyocytes. Front. Cell. Dev. Biol..

[60] Yang Z (2022). Retinoic acid inhibits the angiogenesis of human embryonic stem cell-derived endothelial cells by activating FBP1-mediated gluconeogenesis. Stem Cell Res. Ther..

[61] Hao KL (2023). Disturbance of suprachiasmatic nucleus function improves cardiac repair after myocardial infarction by IGF2-mediated macrophage transition. Acta Pharmacol. Sin..

