# Supplementary material for: Thiamine-modified metabolic reprogramming of human pluripotent stem cell-derived cardiomyocyte under space microgravity
Source: Signal Transduct Target Ther. 2024 Apr 8;9:86. doi: 10.1038/s41392-024-01791-7 (PMC10999445; doi:10.1038/s41392-024-01791-7)
Supplement: Supplementary file 1 — Supplemental figures [file 41392_2024_1791_MOESM1_ESM.pdf]

Supplementary Materials for

Thiamine-modified metabolic reprogramming of human pluripotent stem cell-derived cardiomyocyte under space microgravity

Xinglong Han[1, #], Lina Qu[2, #], Miao Yu[1], Lingqun Ye[1], Liujia Shi[2], Guangfu Ye[2], Jingsi Yang[1], Yaning Wang[1], Hao Fan[1], Yong Wang[1], Yingjun Tan[2], Chunyan Wang[2], Qi Li[2], Wei Lei[1], Jianghai Chen[3], Zhaoxia Liu[2], Zhenya Shen[1, *], Yinghui Li[2, *], Shijun Hu[1, *]

Correspondence to: shijunhu@suda.edu.cn, yinghuidd@vip.sina.com, uuzyshen@aliyun.com

This PDF file includes:

supplementary Figures S1 to S3
supplementary Tables S1 to S4
Captions for supplementary Movies S1 & S2

Other Supplementary Materials for this manuscript include the following:

supplementary Movies S1 & S2

Figure. S1

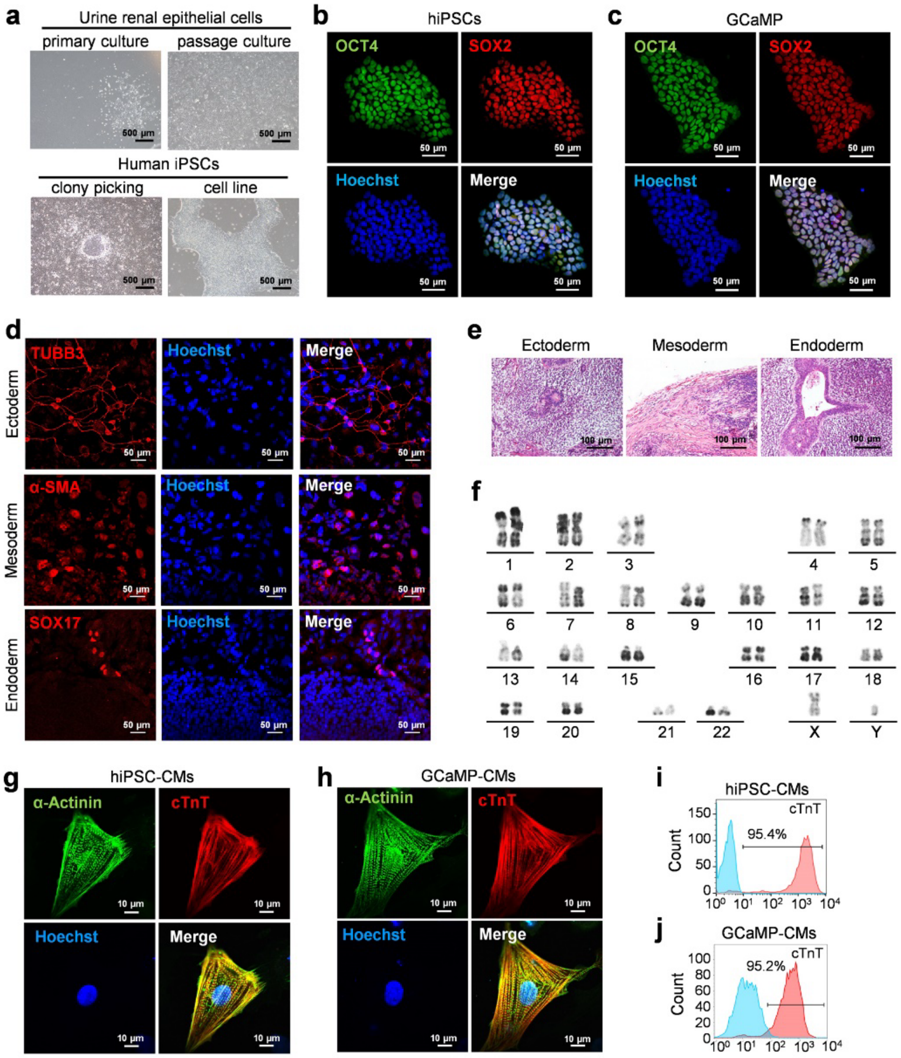

supplementary Figure. S1 Somatic reprogramming and identification. **a** Representative images of urine renal epithelial cells and generated hiPSCs. **b & c** The pluripotent analyses of hiPSCs and GCaMP by immunofluorescent staining. (OCT4, green; SOX2, red; Hoechst 33342, blue). **d** Embryoid body analysis indicated hiPSCs could differentiate into ectoderm (TUBB3), mesoderm (α-SMA), and endoderm (SOX17). **e** Haematoxylin-eosin staining of teratomas revealed hiPSCs could differentiate into ectoderm, mesoderm, and endoderm. **f** Karyotype analysis showed that hiPSCs have normal karyotypes. **g & h** Immunofluorescence staining of hiPSC-CMs and GCaMP-CMs (α-Actinin, green; cTnT, red; Hoechst 33342, blue). **i & j** Flow analyses of cTnT-positive cardiomyocytes.

Figure. S2

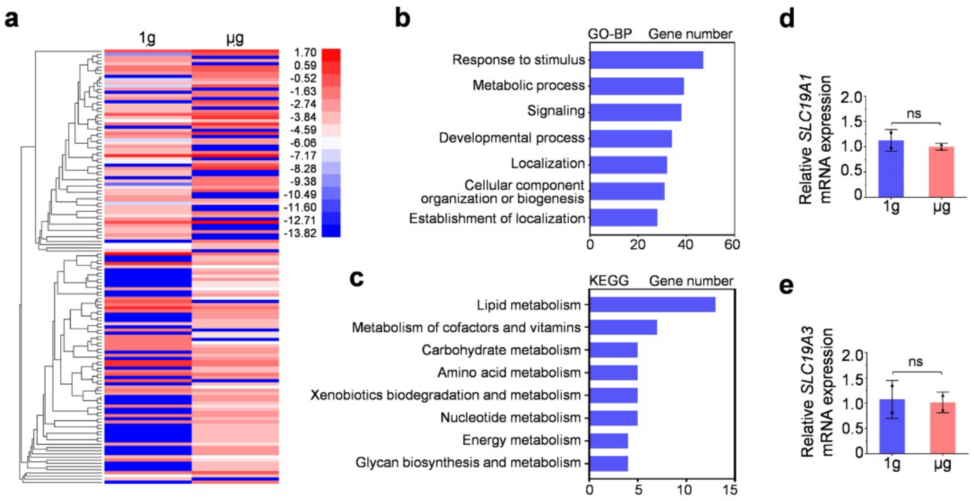

supplementary Figure. S2 Transcriptomic analysis to identify microgravity-induced changes in critical signaling pathways.

a The heat map displayed the differences between 1g and μg. **b** GO-BP Analysis (GO biological process) showed that the differential genes were involved in response to stimulus, metabolic process, signaling, developmental process, localization, and so on. **c** KEGG analysis of metabolic process showed that 8 main categories were identified, including lipid metabolism, metabolism of cofactors and vitamins, carbohydrate metabolism, amino acid metabolism, xenobiotics biodegradation and metabolism, nucleotide metabolism, energy metabolism and glycan biosynthesis and metabolism. The relative mRNA expression of *SLC19A1* (d) and *SLC19A3* (e). Data are presented as mean ± SEM; ns: not significant.

Figure. S3

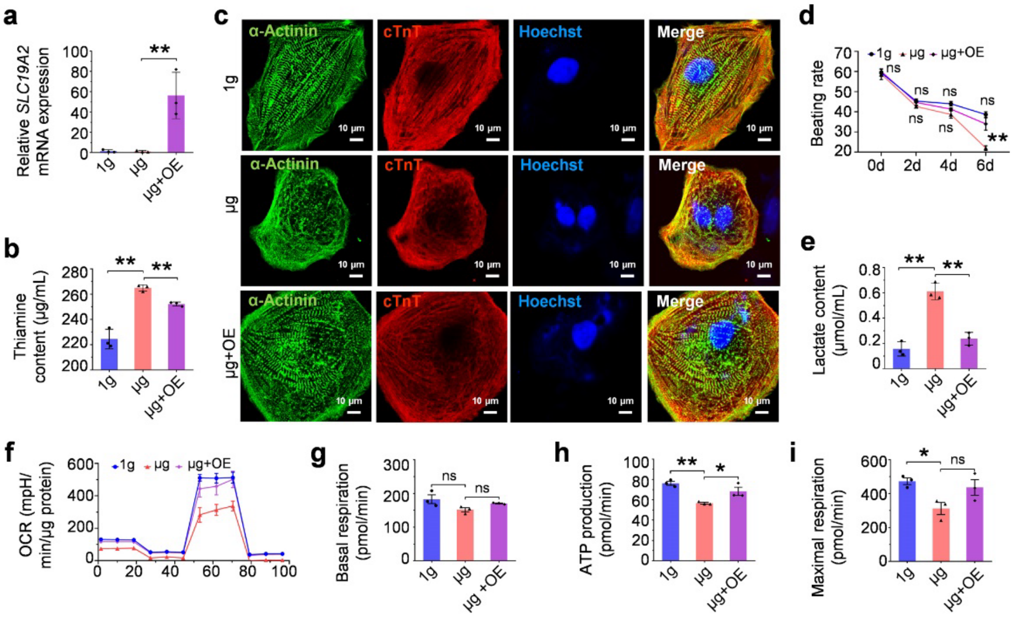

supplementary Figure. S3 Gain-of-function study of *SLC19A2* in cardiomyocytes under simulated-microgravity condition. After infected with *SLC19A2* (OE) or scramble lentivirus, the cardiomyocytes were then treated by simulated microgravity (μg) for 6 days. **a** The relative mRNA expression was detected to ensure *SLC19A2* overexpression. **b** The thiamine content in the cell culture supernatant of hiPSC-CMs under 1g, μg, and μg+OE conditions. **c** Immunofluorescence staining of cytoskeleton proteins in hiPSC-CMs under 1g, μg, and μg+OE group (α-Actinin, green; cTnT, red; Hoechst 33342, blue). **d** The beating rate analyses of GCaMP-CMs in 1g, μg, and μg+OE groups. **e** The analyses of lactate content in cell supernatant of hiPSC-CMs under indicated conditions. **f** Representative OCR traces of hiPSC-CMs in indicated groups were obtained using a Seahorse XFe24 Analyzer. Quantification of basal respiration (g), ATP production (h) and maximal respiration (i). Data are presented as mean ± SEM; *p<0.05, **p<0.01, ns: not significant.

supplementary Table S1. The information of primers.

Targets	Primer sequence (5'-3')
h18S	F-GTAACCCGTTGAACCCCATT
	R-CCATCCAATCGGTAGTAGCG
hMYH6	F-CAAGAGCCGTGACATTGGTG
	R-AGGTTGGCAAGAGTGAGGTT
hMYH7	F-ACCTGTCCAAGTTCCGCAAG
	R-TCATTCAAGCCCTTCGTGCC
hTNNT2	F-ACCAAAGCCCAGGTCGTTC
	R-CAGCGCCTGCAACTCATTC
hTNNI1	F-GCTCCACGAGGACTGAACAA
	R-CTTCAGCAAGAGTTTGCGGG
hTNNI3	F-CCTCCAACTACCGCGCTTAT
	R-CTGCAATTTTCTCGAGGCGG
hRYR2	F-AGCCAGTGTCATCCACCAAC
	R-ACTGATCACAGGTGGCTGAA
hATP2A2	F-TCTCCTTGCCCGTGATTCTC
	R-AACGGCCAGGAAATCCCATC
hSLC8A1	F-TTGAATGAGCTTGGTGGCTT
	R-CCTCTTCCTCTTTGCTGGTC
hCACNA1C	F-CATGCTCACGGTGTTCCA
	R-TCCTACGGCATCATTGACC
hSLC19A2	F-TGACCGAGAGGGAGGTCTTCA
	R-GGTAGTCTGTGGCAAGGAACA
hSLC19A1	F-CTGAGCAGGATGGTGCCCT
	R-TCTCGTTCGTGACCTGCTCC
hSLC19A3	F-CTGACCAGTGCAGAGATAACAA
	R-GATGACTGGCTTGTAGCGGA
mSLC19A2	F-GGTGGAAGAACCGGAGGA
	R-GAAATAGCCGCAGGTGGACA
F, forward; R, reverse.	

supplementary Table S2. The information of antibodies.

**Antibody names**	**Company, Catalog #**	**Dilution**
Oct-3/4 (C-10)	Santa Cruz, sc-5279	1:200 for IF
SOX17	Abcam, ab84990	1:200 for IF
α-Actinin	Abcam, ab9465	1:200 for IF
cTnT	Proteintech, 15513-1-AP	1:200 for IF and FCM
α-SMA	Sigma, A7811	1:200 for IF
Sox2	Santa Cruz, sc-17320	1:200 for IF
TUBB3	Proteintech, 66375-1-Ig	1:200 for IF
TNNI1	Proteintech, 16102-1-AP	1:1000 for WB
TNNI3	Proteintech, 21652-1-AP	1:1000 for WB
SLC19A2	BBI, D221229	1:1000 for WB
Alexa Fluor 594 Anti-mouse	Jackson lab, 715-585-151	1:1000 for IF
Alexa Fluor 594 Anti-rabbit	Jackson lab, 711-585-152	1:1000 for IF and FCM
Alexa Fluor 488 Anti-mouse	Jackson lab, 715-545-151	1:1000 for IF
Alexa Fluor 488 Anti-rabbit	Jackson lab, 711-545-152	1:1000 for IF
Alexa Fluor 647 Anti-WGA	Thermo, W32466	1:1000 for IF
Immunofluorescence (IF); Western blot (WB); Flow cytometry (FCM).		

supplementary Table S3. Differential metabolites between 1g & µg group (related to Figure 2).

Negative iron metabolites												
ID	Name	Formula	Molecular Weight	RT[1]	m/z	MG-1	MG-2	1G-1	1G-2	FC	log2FC	P value
Com_111_neg	LPC 18:2	C26 H50 N O7 P	565.34094	9.014	564.3338	739767223.6	754211137.8	1290888285	1234675883	0.591542	-0.75745	0.010715
Com_2305_neg	LPC 17:1	C25 H50 N O7 P	553.33921	9.139	552.3319	16398410.96	14521292.07	27586965.59	29396833.42	0.542605	-0.88203	0.027556
Com_4710_neg	LPC 22:4	C30 H54 N O7 P	617.37329	9.416	616.3663	3656108.61	4335242.255	8948175.725	10322042.36	0.4147	-1.26986	0.016934
Com_4956_neg	LPC 22:6	C30 H50 N O7 P	613.34229	8.893	612.3352	4193123.664	3835119.246	8280436.664	9202958.807	0.459192	-1.12283	0.008603
Com_7949_neg	(1E,4Z,6E)-5-hydroxy-1,7-bis(4-hydroxyphenyl)hepta-1,4,6-trien-3-one	C19 H16 O4	308.10838	5.311	307.1011	627548.8928	686150.8728	1063895.858	1245320.952	0.568894	-0.81377	0.042625
Com_8597_neg	2-Hydroxy-2-methylbutanedioic acid	C5 H8 O5	148.03676	6.745	341.0717	2538963.354	1988377.449	810994.1734	1069276.116	2.407814	1.267724	0.042407
Com_9497_neg	4-Aminoindole	C8 H8 N2	132.06914	5.655	309.1365	1755175.549	1752992.611	1112612.735	1069663.11	1.607573	0.684884	0.026226

Positive iron metabolites												
ID	Name	Formula	Molecular Weight	RT[1]	m/z	MG-1	MG-2	1G-1	1G-2	FC	log2FC	P value
Com_12932_pos	Lysopa 16:0	C19 H39 O7 P	410.24454	8.81	411.2517	1484280.225	1339449.562	3330310.357	3214671.481	0.431434	-1.21279	0.022978
Com_1531_pos	PC (16:2e/4:0)	C28 H54 N O7 P	547.36541	9.817	548.3725	45286292.07	48462712.82	91240441.44	78267718.13	0.553065	-0.85448	0.048077
Com_2998_pos	PC (18:4e/4:0)	C30 H54 N O7 P	571.36548	9.43	572.3727	17181657.77	19471496.56	35453759.43	38366866.07	0.496516	-1.01009	0.018412
Com_401_pos	Thiamine	C12 H16 N4 O S	264.10489	1.524	265.1122	491219675.1	544441461.9	45844080.61	52100674.57	10.57393	3.40244	0.001536
Com_6621_pos	2-Methoxyestradiol (2-MeOE2)	C19 H26 O3	302.18873	7.479	303.196	5980605.545	5663813.645	11930148.98	11583650.67	0.495216	-1.01387	0.006253
Com_8268_pos	Andrographolide	C20 H30 O5	372.19253	7.056	373.1996	1261835.385	1060330.103	8373145.653	6402038.591	0.157167	-2.66963	0.012611

supplementary Table S4. Differentially expressed genes between 1g and μg group (related to Figure S2).

gene_id	gene_name	gene_locus	MG_FPKM	1G_FPKM	FC	log2FoldChange	pvalue
ENSG00000185864	NPIPB4	16:21834569-21880827	2.793061	0.908279	14.36020737	3.844004678	3.35E-21
ENSG00000145040	UCN2	3:48561718-48563781	1.014929	0.0001185	125.3489304	6.969805876	5.36E-11
ENSG00000284292	AC004922.1	7:99325879-99394653	0.256663	7.362374	0.031303803	-4.997518252	9.56E-11
ENSG00000274600	RIMBP3B	22:21383374-21389478	0	0.1623285	0.002372137	-8.71959719	2.2E-10
ENSG00000135482	ZC3H10	12:56118260-56127514	1.3516185	0.674982	7.493347426	2.905610343	0.00000737
ENSG00000278299	TBC1D3C	17:38057693-38068592	0.0050375	0.274311	0.037833618	-4.72418746	0.0000114
ENSG00000155269	GPR78	4:8558725-8619761	0.0145125	0.2192285	0.089069208	-3.488929416	0.0000189
ENSG00000285404	Z82190.2	22:31754899-31956243	0.2006625	0	159.1970624	7.314669904	0.0000207
ENSG00000254692	AL136295.1	14:24189157-24213473	0	1.3762725	0.000641397	-10.60649567	0.0000332
ENSG00000250506	CDK3	17:76000906-76005999	1.837921	0.4404205	5.478956897	2.453901254	0.0000441
ENSG00000255439	AC135050.2	16:31083439-31094956	0.9884995	0	996.2876937	9.960418593	0.0000514
ENSG00000080293	SCTR	2:119439843-119525301	0.0082445	0.229647	0.048523229	-4.365180642	0.000114704
ENSG00000214736	TOMM6	6:41787662-41789898	10.09398	0.810679	10.68959501	3.418135291	0.000178105
ENSG00000221947	XKR9	8:70669365-70790371	0.0986415	0.371916	0.141519003	-2.820932308	0.000179192
ENSG00000269955	FMC1-LUC7L2	7:139341360-139422599	0.6893965	0	490.7087824	8.938723282	0.000216866
ENSG00000285188	AC008397.2	19:18207961-18255419	0.145013	0.001792	38.93077573	5.282839187	0.000318943
ENSG00000258691	AL355102.2	14:96204844-96263929	0	0.4130295	0.009205409	-6.763302473	0.000318949
ENSG00000248672	LY75-CD302	2:159771851-159904710	0.0609405	0	152.2398759	7.25020248	0.000327444
ENSG00000214447	FAM187A	17:44899712-44905390	0.673504	0.001399	134.2790458	7.06909038	0.000375092
ENSG00000286185	AC242842.3	1:149390623-149556361	0.696867	0.292766	9.4272555	3.236837829	0.000405255
ENSG00000253797	UTP14C	13:52024691-52033600	0.2160395	4.3182685	0.05078494	-4.299455466	0.00052798
ENSG00000244607	CCDC13	3:42708269-42773253	0.079811	0.5497045	0.154953958	-2.690088486	0.000580591
ENSG00000107165	TYRP1	9:12685439-12710290	0	0.1452725	0.009035478	-6.790183281	0.000588562
ENSG00000221874	ZNF816-ZNF321P	19:52928475-52962823	0.1975235	0	101.2457498	6.661717536	0.000601094
ENSG00000105851	PIK3CG	7:106865278-106908980	0.0443185	0	105.93465	6.727030745	0.000631224
ENSG00000198211	AC092143.1	16:89919165-89936092	0.398989	0.000051	96.46746204	6.591970506	0.000792501
ENSG00000164398	ACSL6	5:131949973-132012243	0.0375165	0	93.31854718	6.544091942	0.001284807
ENSG00000196844	PATE2	11:125776113-125778819	0.1261725	0	91.89069747	6.521846913	0.001387682
ENSG00000066230	SLC9A3	5:469397-524332	1.309474	0.286601	8.214381256	3.038151909	0.001677195
ENSG00000257390	AC023055.1	12:55757275-55827546	0.4576295	3.044732	0.061389445	-4.02586556	0.001730039
ENSG00000184986	TMEM121	14:105526583-105530202	1.4226255	0.373171	12.10723409	3.597797412	0.001869758

ENSG00000277632	CCL3	17:36088256-36090169	0.2282845	1.4563075	0.199001617	-2.329147943	0.002085076
ENSG00000145832	SLC25A48	5:135579202-135889770	0.084848	0	84.98029567	6.409056458	0.002323254
ENSG00000272414	FAM47E-STBD1	4:76251721-76311129	0.518876	4.15494	0.075558717	-3.726257976	0.00234991
ENSG00000142677	IL22RA1	1:24119771-24143140	0.0689985	0	79.90114759	6.320144319	0.00244617
ENSG00000284776	AL121900.2	20:18567453-18744216	0	0.6601605	0.00501372	-7.639902815	0.002715254
ENSG00000286237	ARMCX5-GPRASP2	X:102712495-102753530	0.0750375	0.422995	0.177527855	-2.493882687	0.00297617
ENSG00000226124	FTCDNL1	2:199760544-199851173	0.538168	0.062904	5.606240025	2.487033513	0.00302294
ENSG00000214415	GNAT3	7:80458671-80512020	0.1574495	0	79.49192137	6.312736344	0.003138457
ENSG00000257921	AC025165.3	12:57772660-57797554	0.116117	2.1042785	0.016257715	-5.942731679	0.003222778
ENSG00000270617	URGCP-MRPS24	7:43866558-43906589	3.63623	0.062048	26.85557373	4.747149638	0.003679615
ENSG00000263020	AL662899.2	6:31666102-31673546	2.652612	0.161349	23.57133915	4.558961819	0.004055188
ENSG00000163687	DNASE1L3	3:58192257-58214697	0	0.108793	0.013615974	-6.198555959	0.004169239
ENSG00000143882	ATP6V1C2	2:10721100-10785110	0.163626	0.077761	6.25338028	2.644636253	0.004180802
ENSG00000185198	PRSS57	19:685546-695498	0	0.1502535	0.014269833	-6.130887753	0.005038333
ENSG00000121211	MND1	4:153344649-153415118	0.499536	0.052603	8.098907499	3.017727309	0.005061349
ENSG00000188305	PEAK3	19:2274631-2282175	0	0.062046	0.014336278	-6.124185649	0.005332664
ENSG00000250709	CCDC169-SOHLH2	13:36168794-36297842	0	0.1664255	0.006943804	-7.170058001	0.006023192
ENSG00000173110	HSPA6	1:161524540-161526894	16.524116	2.8916385	5.227643951	2.386160884	0.006026498
ENSG00000196616	ADH1B	4:99304971-99352760	0.036127	0	72.57902393	6.18148075	0.006187649
ENSG00000125879	OTOR	20:16748358-16770062	0.1682	0	77.07849279	6.268256456	0.00638585
ENSG00000265118	AC134669.1	17:31169683-31330447	0.0594265	0.286026	0.034166379	-4.871278818	0.006586485
ENSG00000166292	TMEM100	17:55719627-55732121	0.3210915	0.042968	7.005351228	2.808457385	0.006956576
ENSG00000186452	TMPRSS12	12:50842920-50887884	0.0645545	0	90.40717526	6.498365373	0.007245307
ENSG00000254737	OR10G4	11:124012997-124018732	0.0743665	0	68.30491913	6.093917576	0.007892581
ENSG00000105675	ATP4A	19:35550043-35563658	0.0406515	0	68.3049392	6.093918	0.007895691
ENSG00000188050	RNF133	7:122697712-122699156	0	0.109705	0.013867658	-6.17213198	0.008220182
ENSG00000074803	SLC12A1	15:48168673-48304078	0.1132505	0.016006	6.945548657	2.796088663	0.008412167
ENSG00000285938	AC072022.2	3:187743686-187746028	0.02075	0.212948	0.123904711	-3.012697053	0.009003713
ENSG00000170465	KRT6C	12:52468516-52473805	0	0.059125	0.01595162	-5.970153286	0.009494514
ENSG00000070019	GUCY2C	12:14612632-14696599	0.1000255	0.0020805	17.85407109	4.15818117	0.009675883
ENSG00000249590	AC004832.3	22:30409255-30428990	0.626253	0.036252	23.22669067	4.537711709	0.010196596
ENSG00000248592	STIMATE-MUSTN1	3:52833121-52897562	0.2349015	0.0549095	6.298182476	2.654935557	0.010253027
ENSG00000182256	GABRG3	15:26971181-27541984	0.0526315	0.0446215	0.141305081	-2.82311475	0.010949851
ENSG00000212123	PRR22	19:5782960-5784746	0.340219	0.041175	6.046353003	2.59606521	0.01164482

ENSG00000205704	LINC00634	22:41952174-41958933	0.181305	0.012495	11.32629333	3.501603893	0.011955855
ENSG00000284057	AP001273.2	11:93741664-93812378	0	0.087487	0.009003065	-6.795368093	0.012107943
ENSG00000186907	RTN4RL2	11:57460528-57477534	0	0.076161	0.013955141	-6.163059475	0.012261896
ENSG00000099834	CDHR5	11:616565-626078	0	0.0464175	0.016762043	-5.898658225	0.013037655
ENSG00000174145	NWD2	4:37244743-37449463	0.01746	0	59.1607767	5.886569088	0.013226755
ENSG00000180934	OR56A1	11:6019336-6034338	0.0296555	0.0606885	0.102148895	-3.291254502	0.014282596
ENSG00000125850	OVOL2	20:17956979-18059188	0.0972375	0	64.46624431	6.010472032	0.014889064
ENSG00000169181	GSG1L	16:27787528-28063714	0.025679	0	55.28762026	5.78888457	0.01514198
ENSG00000273703	HIST1H2BM	6:27815044-27815424	0.3362295	0	62.59885843	5.968064443	0.016528127
ENSG00000152705	CATSPER3	5:134908830-135011696	0.070225	0.2759735	0.151643264	-2.721246683	0.01680699
ENSG00000196169	KIF19	17:74326210-74355820	0.006213	0.092807	0.108822731	-3.199948159	0.0210315
ENSG00000260371	AC026464.3	16:69335091-69356306	0.082827	0.4606435	0.193369734	-2.37056609	0.021710579
ENSG00000144130	NT5DC4	2:112721486-112742879	0.0893335	0	55.9081197	5.80498592	0.022332551
ENSG00000188691	OR56A5	11:5967177-5968494	0.006512	0.207268	0.106236097	-3.234654044	0.022724167
ENSG00000197540	GZMM	19:544034-549924	0.022867	0.2122715	0.108424335	-3.205239498	0.022967274
ENSG00000178403	NEUROG2	4:112513516-112516180	0.1216265	0.0153405	7.282474779	2.8644288	0.023203952
ENSG00000171133	OR2K2	9:111327483-111330183	0.1027005	0	51.4196958	5.68424917	0.023370766
ENSG00000257767	AC002996.1	12:111753890-111791418	0.295723	0	90.95450263	6.507073154	0.023554263
ENSG00000119283	TRIM67	1:231162112-231221556	0.0040815	0.0329485	0.168144659	-2.572225142	0.023718418
ENSG00000187908	DMBT1	10:122560665-122643740	0.0346295	0.004289	7.078578243	2.823459619	0.024198595
ENSG00000019991	HGF	7:81699010-81770438	0	0.0316455	0.019249722	-5.699018585	0.024796905
ENSG00000185681	MORN5	9:122159908-122200088	0.050874	0.448882	0.160311989	-2.641045771	0.026942447
ENSG00000148734	NPFFR1	10:70247329-70283676	0.013133	0	50.62526045	5.66178552	0.027277438
ENSG00000125998	FAM83C	20:35285731-35292425	0.0382365	0	50.6252069	5.661783994	0.027310862
ENSG00000101445	PPP1R16B	20:38805697-38923024	0.0065195	0.043183	0.185508015	-2.430446576	0.027433526
ENSG00000198785	GRIN3A	9:101569352-101738580	0	0.014609	0.019876243	-5.652811125	0.027629326
ENSG00000157766	ACAN	15:88803442-88875354	0.0305785	0.001979	7.507456399	2.908324191	0.028915997
ENSG00000268533	AC003002.2	19:57394183-57438457	0	0.35545	0.012218576	-6.354780088	0.029241863
ENSG00000249773	AC092647.5	7:55887277-55956500	0.085263	0.568374	0.137108833	-2.866606579	0.029965701
ENSG00000164303	ENPP6	4:184088706-184221230	0.0470515	0.093793	0.192038421	-2.380533114	0.03133708
ENSG00000108878	CACNG1	17:67044554-67056797	0.075748	0	50.41777585	5.655860571	0.031750846
ENSG00000131969	ABHD12B	14:50872053-50904970	0	0.0425295	0.020011813	-5.643004333	0.032118344
ENSG00000118513	MYB	6:135181308-135219173	0	0.0300205	0.021301037	-5.55293253	0.033660851
ENSG00000165349	SLC7A3	X:70925579-70931125	0.0425165	0	46.75454967	5.547034855	0.034058108

ENSG00000165966	PDZRN4	12:41188448-41574724	0	0.02797	0.023120442	-5.434687192	0.034090751
ENSG00000124203	ZNF831	20:59123381-59259113	0.0092595	0	46.94995575	5.553051893	0.034328725
ENSG00000204702	OR2J1	6:29099657-29102701	0.0706425	0	46.94996515	5.553052182	0.034346623
ENSG00000179520	SLC17A8	12:100357079-100422059	0.04497	0.004737	8.647435645	3.112272372	0.034655891
ENSG00000168658	VWA3B	2:98087116-98313299	0.047413	0	51.85370275	5.696375107	0.034743343
ENSG00000176230	OR4K17	14:20110739-20122199	0	0.061842	0.022952491	-5.445205464	0.034745025
ENSG00000137707	BTG4	11:111467526-111512354	0.005829	0.098454	0.087077677	-3.521553268	0.034820034
ENSG00000250424	AC004691.2	7:30852273-30923812	0	0.115291	0.013430452	-6.218348351	0.034955304
ENSG00000063015	SEZ6	17:28954901-29006440	0.0893745	0	46.55752605	5.540942494	0.036109073
ENSG00000196860	TOMM20L	14:58395928-58409771	0.157271	0.072184	5.473988019	2.452592277	0.036156409
ENSG00000168004	PLAAT5	11:63461404-63491194	0	0.032313	0.020717671	-5.592994348	0.036692126
ENSG00000242515	UGT1A10	2:233636454-233773305	0.044806	0	47.14877367	5.559148342	0.036707603
ENSG00000183134	PTGDR2	11:60850933-60855950	0	0.0406295	0.018985409	-5.718965081	0.037163117
ENSG00000141485	SLC13A5	17:6684713-6713567	0.0460585	0.0032975	9.66852938	3.273296467	0.037386164
ENSG00000165970	SLC6A5	11:20599400-20659285	0.0254635	0	73.79940144	6.20553721	0.03922423
ENSG00000154227	CERS3	15:100400395-100544995	0.1073315	0	73.79944405	6.205538043	0.039243545
ENSG00000126246	IGFLR1	19:35738801-35742453	0.2846665	0	73.79947658	6.205538679	0.039258318
ENSG00000235718	MFRP	11:119338942-119346705	0.033871	0	56.37659698	5.817024491	0.03973228
ENSG00000285130	AL358113.1	9:69035747-69255187	2.3170135	0.4628255	6.348316313	2.666374014	0.039926946
ENSG00000241186	TDGF1	3:46574534-46582457	0.3766215	0.054485	6.50316218	2.701141403	0.039960704
ENSG00000111834	RSPH4A	6:116616479-116632985	0.07344	0.006679	6.953182426	2.797673441	0.040180456
ENSG00000256514	AP003419.1	11:67351572-67373584	0.3744045	0.000001	21.74709167	4.442750571	0.040945394
ENSG00000092200	RPGRIP1	14:21287939-21351301	0.0256325	0	42.49147278	5.409101444	0.04314077
ENSG00000111780	AL021546.1	12:120438198-120460006	0.570505	0.086702	5.373712546	2.42591915	0.043934256
ENSG00000186487	MYT1L	2:1789113-2331664	0.062315	0	69.51337245	6.119218634	0.046084367
ENSG00000185758	CLDN24	4:183321764-183322426	0.0170045	0.190511	0.093995794	-3.411259988	0.046412336
ENSG00000124191	TOX2	20:43914852-44069616	0	0.0312535	0.025484372	-5.294243412	0.046850993
ENSG00000173626	TRAPPC3L	6:116494989-116545610	0.033641	0	42.29438597	5.402394272	0.047647039
ENSG00000182177	ASB18	2:236194872-236264409	0.0219475	0	42.2943578	5.402393311	0.047670946
ENSG00000107593	PKD2L1	10:100288149-100330264	0.032778	0	42.29431224	5.402391757	0.047709644
ENSG00000113946	CLDN16	3:190322541-190412143	0.0569895	0.0054145	7.902751283	2.982355004	0.048365456
ENSG00000213714	FAM209B	20:56533246-56536520	0.321245	0.0509045	6.008987655	2.587121958	0.048779919
ENSG00000152969	JAKMIP1	4:6026203-6200591	0	0.0362015	0.023470092	-5.413032673	0.048918977
ENSG00000166006	KCNC2	12:75040077-75209868	0.0189385	0	43.28946366	5.435944021	0.048968036

ENSG00000175544	CABP4	11:67452406-67460313	0.042236	0	43.28946978	5.435944225	0.048971798
ENSG00000197430	OPALIN	10:96343221-96359365	0.028463	0	46.13947944	5.527929822	0.049060588
ENSG00000188710	QRFP	9:130892702-130896812	0.052405	0	43.07657532	5.428831652	0.049114873
ENSG00000148156	ACTL7B	9:108854589-108856967	0	0.0451765	0.02177087	-5.521457117	0.049656203

supplementary Movie S1. Video recording of GCaMP-CMs with fluorescence indicative in 1g group (related to Figure 1).

supplementary Movie S2. Video recording of GCaMP-CMs with fluorescence indicative in μg group (related to Figure 1).
